# Predictors of low birth weight and 24-hour perinatal outcomes at Muhimbili National Hospital in Dar es Salaam, Tanzania: a five-year retrospective analysis of obstetric records

**DOI:** 10.11604/pamj.2018.29.220.15247

**Published:** 2018-04-23

**Authors:** Benjamin Anathory Kamala, Andrew Hans Mgaya, Matilda Michael Ngarina, Hussein Lesio Kidanto

**Affiliations:** 1 Department of Obstetrics and Gynaecology, Muhimbili National Hospital, Dar es Salaam, Tanzania; 2 Department of Health Science, University of Stavanger, Stavanger, Norway; 3 Department of Women’s and Children’s Health/International Maternal and Child Health, Uppsala University, Uppsala, Sweden

**Keywords:** Low-birth-weight, perinatal outcomes, intra-uterine growth restriction

## Abstract

**Introduction:**

The global prevalence of low birth weight (LBW) is 16%, representing more than 20 million infants worldwide, of which 96% are born in low-income countries. This study aimed to determine the prevalence, predictors and perinatal outcomes of LBW newborns.

**Methods:**

We conducted a retrospective analysis of data obtained from the hospital's obstetric and neonatal database. Descriptive statistics and multivariate logistic regression were performed with 95% confidence intervals (CI).

**Results:**

The prevalence of LBW was 21% (n = 8,011) and two-thirds of these were delivered at term. Seven percent of newborns were stillbirths and 2% died within 24hrs after birth. Logistic regression revealed that primigravida and grand multiparity were associated with LBW (OR: 1.25, 95%CI: 1.15-1.37; and OR: 1.21, 95%CI: 1.01-1.25, respectively). Having <4 antenatal care (ANC) visits was associated with increased odds of LBW (OR: 1.74, 95%CI: 1.59-1.87). Regression models revealed an independent association between LBW and increased odds of stillbirths (OR = 7.20, 95%CI 6.71-7.90), low Apgar score (OR = 3.42, 95%CI: 3.12-3.76) and early neonatal deaths (OR = 1.82, 95%CI: 1.51-2.19).

**Conclusion:**

The prevalence of LBW was high and was associated with extreme maternal age groups, grand multiparity, low maternal education, low number of ANC visits and obstetrics risks factors and complications. Both LBW and prematurity were independently associated with poor perinatal outcome. Future interventions should focus on improving the quality of ANC and integrating peripartum emergency obstetric and neonatal care.

## Introduction

The World Health Organization (WHO) defines low birth weight (LBW) as a newborn weighing is less than 2,500 grams, with the measurement taken within the first hours of life, before significant postnatal weight loss has occurred [1]. Globally, the prevalence of LBW is 14% and, according to the WHO and the World Bank, more than 96% of LBW newborns are born in developing countries [1,2]. Hospital-based studies in Sub-Saharan Africa indicated a wide variation in inter-country prevalence of LBW, from 9% in Rwanda [3,4] to 31% in Sudan [2]. In Tanzania, despite regional variation and an increase in population from 34.4 million in 2002 to 44.9 million in 2012, the prevalence of LBW has been in decline; from 13% to 7% over nearly the same period (from 2000 to 2010) [5]. LBW has been strongly associated with poor health outcomes during the neonatal period, infancy and adulthood [2,6], regardless of race and socioeconomic status [7,8]. For example: a study conducted in the USA investigating births among different races showed that LBW substantially increased the risk of stillbirth, irrespective of race [8]. Furthermore, approximately every ten seconds, an infant in a developing country dies as a result of a disease or infection that can be attributed to LBW [7]. A recent epidemiological study [9] addressed the association between LBW and medical conditions, including metabolic syndrome (a cluster of conditions that include high blood pressure, high blood sugar, excessive body fat around the waist) and abnormal cholesterol or triglyceride levels. A meta-analysis of the literature also found that LBW is associated with an increased risk of heart disease, stroke and diabetes in adulthood [10,11].

Having LBW imposes a 20-times higher likelihood of death during infancy compared to newborns who had normal birth weight [1]; therefore, LBW is associated with high infant mortality and low life expectancy and also predicts prevalence of disability and educational achievement in childhood and adulthood [9,10]. As a health indicator, LBW has been used as a good summary measure to indicate a wide range of multifaceted public health problems that include health inequality, long-term maternal malnutrition, ill health, hard physical work and poor care during pregnancy [9]. Female newborns and first-born infants are generally lighter than their counterparts [12]. Evidence from various studies and the WHO report highlighted modifiable and non-modifiable predictors of risk factors for LBW, including female sex, primiparity, multiple pregnancies, low socio-economic status (SES), nutritional insufficiency and short stature [12-14]. Other identified factors were higher education, single motherhood, maternal complications and lack of antenatal care (ANC) [6]. The most recent national estimates are drawn from TDHS 2010 [5] as the 2015 full report has not yet been released [15]. The absence of recent estimates of LBW hinders adequate assessment of the demands and appropriate allocation of health resource for managing LBW newborns in hospital settings. Local needs assessment enables the successful implementation of interventions for achieving Sustainable Development Goals that are essential for a healthy start for a newborn, from the pregnancy period to safe childbirth. Prevention and adequate management of LBW also make an important contribution to the third Sustainable Development Goal (SDG 3), which focuses on the reduction of neonatal and under-5 mortality [16]. Therefore, this study aimed to determine the prevalence and predictors of LBW and its 24-hour delivery outcomes at Muhimbili National Hospital in Dar es Salaam (MNH).

## Methods

**Study design, sampling and participants**: A descriptive retrospective study to determine the prevalence and predictors of LBW was undertaken at MNH from February 2010 to September 2015. During the study period there were 48,918 deliveries, the details of which were prospectively recorded in the delivery book and then entered on the database. Out of these, 9,819 births were excluded due to their having incomplete records or a gestational age of less than 28 weeks. Thus, 39,099 deliveries were included in the analyses.

**Study site**: MNH is a teaching hospital of Muhimbili University of Health and Allied Sciences (MUHAS). It is situated in the main commercial city of Dar es Salaam, which has a population of about 5 million people and an annual population growth rate of 5.6% [17]. Annual deliveries total approximately 10,000. The hospital provides comprehensive emergency obstetric care. Currently, the Neonatal Intensive Care unit provides continuous positive air pressure (CPAP) and surfactant for preterm babies. However, this was only introduced in 2014. The gestational age cutoff for resuscitation is 28 weeks. Varying management protocols for mothers and babies have been described in previous papers in this setting [18]. Qualified data entry clerks have been digitally entering data, including maternal and neonatal outcomes, prospectively from midwifery books since 1998 [18,19]. Maternal and neonatal diagnoses are based on doctors' records in the case notes. Neonatal deaths reflect only babies who were born at MNH and were admitted to the neonatal unit.

**Study variables**: The variables included were: maternal age in complete years; education level; marital status; ANC attendance; and pregnancy and delivery complications, all of which were taken from their ANC card on admission. Others were: parity; gestational age; and area of residence. Gestational age was estimated from Naegele's rule of last normal menstrual period (LNMP) and a first trimester ultrasound. Gestational age was then dichotomised into preterm and term. Birth weight was measured within the first hour of life and dichotomised as LBW and normal birth weight, according to the WHO definitions (if less than 2,500 grams). In this paper we considered LBW and normal birth weight against various predictors, while for gestational age we added a regression model to control for its potential confounding effect. Outcome measures were birth weight, stillbirth (a baby born with no signs of life at or after 28 weeks' gestation) and the status of the baby after 24 hours, recorded as normal, admitted to neonatal unit, or dead.

**Data management and analysis**: Data were retrieved from the obstetric database, cleaned and analysed using Statistical Package for the Social Sciences (SPSS v22, IBM, Armonk, NY, USA). The characteristics of the study participants were calculated using descriptive statistics. Bivariate analyses were completed by comparing each of the variables with birth weights, and by conducting χ^2^ analysis and odds ratio (OR) calculations with 95% confidence intervals (CI). Regression analysis was completed to identify and quantify predictors of LBW and significance was set at a p-value of less than 0.05.

**Ethical clearance and ethical consideration**: Ethical clearance was sought from the MNH Institutional Review Board (IRB) to use the electronic obstetric database (approval number MNH/IRB/I/2016/26). The board has approved the publication of these findings. Anonymity and confidentiality of the information was guaranteed and as data did not include maternal identity, such as names, consent was not needed.

## Results

**Maternal-related characteristics**: We analysed 39,099 deliveries that met the inclusion criteria. The majority (81.6%) of the participants were aged between 20-35 years. Ninety-seven percent of women were ever-married (Table 1). One-third of the mothers attended ANC less than four times.

**Table 1:** Percentage distribution of factors associated with the low birth weight of babies born at MNH from February 2010 to September 2015

Variable	Values	Frequency	Percentage (%)
Maternal age (years)	<20	2,150	5.5
	20–35	31,905	81.6
	>35	5,005	12.8
Parity	Prime	5,357	13.7
	2–4	22,404	57.3
	Grand multiparity	11,339	29
ANC visits	<4	11,261	28.8
	4^+^	27,956	71.5
ANC maternal problem	None	25,532	65.3
	Anaemia	1,095	2.8
	Hypertension	2,385	6.1
	HIV	1,056	2.7
	Multiple pregnancy	274	0.7
	PROM	274	0.7
	Others	8,524	21.8
Gestational age	Term	36,636	93.7
	Preterm	2,463	6.3
Area of residence	Rural/Semi urban	5,748	14.7
	Urban	33,351	85.3
Education	Primary	22,247	56.9
	Secondary	9,266	23.7
	Post-secondary	7,585	19.4
Marital Status	Never married	1,368	3.5
	Ever-married	37,731	96.5
Sex of the Newborn	Male	20,449	52.3
	Female	18,650	47.7
Apgar Score at 1 Minute	< 7	8,875	22.7
	7^+^	34,133	87.3
Apgar Score at 5 Minutes	< 7	5,826	14.9
	7^+^	33,273	85.1
Birth outcomes	Stillbirth	2,698	6.9
	Alive	36,401	93.1
Neonatal outcomes in 24-hours	Dead	704	1.8
	Alive	38,395	98.2
Birth weight	Low birth weight (< 2,500gm)	8,015	20.5
****	Normal birth weight	31,084	79.5

**Birth outcomes**: The median birth weight was 3,000g, ranging from 700g to 5,900g and the mean (SD) was 2,940g (702g). The prevalence of LBW was 21% (n = 8,011). The prevalence of stillbirths (both fresh and macerated) and very early neonatal deaths was 6.9% and 1.8%, respectively, of the live births who later died, making the total number of deaths about 9%.

**Mode of delivery**: Caesarean section (CS) delivery was performed in 55% (n = 21,504) of all included deliveries, and one-fifth of these were performed electively. Premature deliveries accounted for only 3.9% and 2.4% of all CS and elective deliveries, respectively.

**Birth weights, gestational age and perinatal outcomes**: Gestational age (GA) at delivery ranged from 28 to 43 weeks with the mean and median GA of 38 and 39 weeks, respectively. About 93.6% were term, 6.4% were preterm, and less than 0.1% were post-term deliveries. Of the term pregnancies, 15.0% were small for gestational age (SGA), sometimes referred to as intrauterine growth restriction (IUGR), 82.4% were appropriate for gestational age (AGA) and only 2.6% were large for gestational age (LGA). In both term and preterm categories, LBW was associated with adverse perinatal outcomes, although the rates were much higher among preterm deliveries, as shown in Table 2 below.

**Table 2:** Distribution of birth weight and gestational age in relation to perinatal outcomes among babies born at MNH between 2010 and 2015

	Pre-term				Term			
	Normal BW	LBW	Total	*p*-value	Normal BW	LBW	Total	*p*-value
	*n*=76	*n*=2,387	*n*=2,463		*n*=31,008	*n*=5,628	*n*=36,636	
Apgar Score at 1 min (< 7)	48.70%	64.30%	63.80%	0.005	12.00%	34%	15.10%	0.001
Apgar Score at 5 min (< 7)	36.80%	53.70%	53.20%	0.004	7.60%	24.50%	10.10%	0.001
Stillbirth	18.40%	33.00%	32.50%	0.008	3.40%	14.70%	5.10%	0.001
Early neonatal deaths	10.0%	7.70%	7.80%	0.06	0.80%	2.40%	1.10%	0.001

**Birth weights and mothers' sociodemographic characteristics**: Mothers residing in semi-urban areas had 23% higher odds of delivering an LBW baby compared to those in urban areas (p = 0.001). LBW deliveries were associated with lower levels of education (χ^2^ for the trend 747.3, p = 0.001). LBW deliveries varied with age, whereby adolescents and mothers aged above 35 years were more likely to deliver LBW babies compared to those aged 20-35 years. Also, ever-married mothers had 25% decreased odds of delivering LBW babies compared to never-married mothers (p = 0.001), as shown in Table 3 below.

**Table 3:** Bivariate analysis (unadjusted odds ratio) of factors associated with low birth weight among babies born at MNH from February 2010 to September 2015

Socio-demographic factors		Unadjusted OR	95% Confidence Intervals		*p*-value
			Lower	Upper	
Age groups	<20	1.52	1.38	1.68	0.001
	20 – 35	1			
	35^+^	1.08	1.01	1.16	0.036
Parity	2– 4	1			
	Primiparous	1.31	1.23	1.40	0.001
	Grand multiparity	1.38	1.29	1.49	0.001
ANC visits	<4	3.29	3.13	3.47	0.001
	4^+^	1			
**Maternal related factors**					
ANC health problems	Normal	1			
	Anaemia	2.49	2.19	2.83	0.001
	Hypertension	3.76	3.45	4.11	0.001
	Prev. Scar	0.62	0.58	0.69	0.001
	HIV	1.34	1.15	1.56	0.001
	Multiple pregnancy	5.72	4.23	7.74	0.001
	PROM	4.55	3.61	5.72	0.001
	Others^+^	1.09	0.92	1.28	0.327
Gestational age	Preterm	175.34	139.81	220.91	<0.001
Areas of residence	Semi-urban	1.23	1.12	1.36	0.001
	Urban				
Education level	Primary	2.61	2.43	2.81	0.001
	Secondary	1.72	1.58	1.87	0.001
	College	1			
Marital status	Never married	1.35	1.18	1.54	0.001
	Ever married	1			
Birth outcomes					
Sex of the new-born	Male	1			
	Female	1.19	1.14	1.26	0
Apgar Score at 1 minute	0 – 6	5.64	5.34	5.97	0.001
Apgar Score at 5 minutes	0 – 6	6.13	5.79	6.56	<0.001
Birth outcomes	Stillbirth	7.29	6.71	7.91	0.001
Neonatal Outcomes in 24 hours	Death	5.99	5.04	7.00	0.001

+ Others: Sickle cell disease, recurrent abortion, Rh-negative factors, prev. PPH, and cardiac diseases

**Birthweights and mothers' obstetric histories**: In this study, the prevalence of LBW deliveries was higher among mothers with recorded ANC problems but lower among those with a history of caesarean section. Mothers of LBW babies were more than two times more likely to have a maternal history of anaemia, hypertension, multiple gestation and PROM. Mothers who delivered by caesarean section were 40% less likely to have an LBW delivery in the index delivery (p < 0.001). Primigravida and grand multiparous (> 4 deliveries) mothers had increased odds of delivering an LBW infant compared to mothers with 2-4 children (p<0.001). On ANC attendance, those who attended less than four times were more than 3 times more likely to deliver LBW compared to those who attended four or more times (p < 0.001). A one-week increase in gestational age was associated with 3.4 times higher odds of delivering a normal birth baby (p < 0.001).

**Birthweights and outcomes**: Stillborn babies had an almost 7 times higher chance of being born with LBW (p < 0.001), and those dying within 24 hours were three times more likely to be born with LBW (p < 0.001) as well those with an Apgar score of less than 7 at the first and fifth minute (p < 0.001).

**Multivariate regression analysis**: Two models of multivariate logistic regression were completed. The first model was designed to identify and quantify true predictors of LBW and control for potential confounders, including gestational age dichotomised into preterm and term. The variables were added in two blocks of socio-demographic characteristics and predictors. For maternal age, the adolescent mothers remained at risk of having LBW babies compared to those aged 35 years and above. All ANC problems were associated with LBW, except for HIV infection, which had no effect on LBW and previous caesarean section, which was associated with reduced risk of LBW. Parity of 2-3 children was associated with a reduced risk of LBW compared with primigravida and having 4 or more children. Having attended less than four ANC visits was associated with an increased risk of LBW. In the second model, outcomes were combined into a single block. Stillbirths had increased odds of being LBW babies. Also, LBW babies were more likely to have an Apgar score of less than 7 at the first and fifth minute compared to normal weight babies. Baby girls were marginally at increased risk of being born with LBW compared to boys. The details are shown in Table 4. Separate regression analysis showed that LBW among preterm deliveries was associated with low Apgar score at the first and fifth minute but not stillbirth and early neonatal death (as the rates were higher regardless of birth weight), whereas LBW was associated with increased likelihood of adverse perinatal outcomes among the term deliveries, as shown in Table 5 below.

**Table 4:** Multivariate logistic regression predictors of low birth weight and associated outcomes among babies born at MNH from February 2010 to September 2015

		AOR	95% Confidence Interval		p-value
Model 1: Predictors			Lower	Upper	
Maternal age	<20	1.09	0.99	1.20	0.089
	20-35	1			
	35+	0.96	0.03	1.10	0.558
Parity	1	1.26	1.15	1.37	0.002
	2–3	1			
	4+	1.12	1.01	1.24	0.038
ANC visits	<4	1.74	1.59	1.89	0.001
Education	Primary	2.11	1.72	2.32	<0.001
	Secondary	1.60	1.44	1.79	0.001
	College	1			
Marital status	Never married	1.04	0.96	1.28	0.684
	Ever married	1			
Areas of residence	Semi-urban	1.09	0.96	1.22	0.188
	Urban	1			
Antenatal health problem	Normal	1			
	Anaemia	1.81	1.56	2.12	0.012
	Hypertension	2.81	2.51	3.13	0.001
	Prev. Scar	0.69	0.64	0.75	0.001
	HIV	1.16	0.94	1.27	0.238
	Multiple pregnancy	5.64	3.84	8.25	0.001
	PROM	3.17	2.31	4.23	0.001
Gestational Age	Preterm	147.27	112.71	192.43	<0.001
Areas of residence	Semi-urban	1.09	0.96	1.22	0.188
	Urban	1			
Education	Primary	2.11	1.72	2.32	<0.001
	Secondary	1.60	1.44	1.79	0.001
	College	1			
Marital status	Never married	1.04	0.96	1.28	0.684
	Ever married	1			
Model 2: Associated Outcomes					
Sex of the child	Male	0.68	0.81	1.01	0.068
	Female	1			
Apgar Score at 1 Minute	<7	3.42	3.12	3.76	0.001
Apgar Score at 5 Minutes	< 7	1.26	1.11	1.43	0.001
Birth outcome	Stillbirth	7.29	6.71	7.91	0.001
Neonatal outcome in 24 hours	Death	1.82	1.51	2.19	0.001

**Table 5:** Multivariate logistic regressions analysis of the effect of LBW on adverse perinatal outcomes among term and preterm deliveries at MNH from February 2010 to September 2015

		Apgar at 1 min (< 7)		Apgar at 5 min (< 7)		Stillbirths		Early neonatal death	
		AOR	95% CI	AOR	95% CI	AOR	95% CI	AOR	95% CI
**Preterm**	Normal BW	1		1		1		1	
	LBW	1.65	1.03 –2.64	1.70	1.05 –2.76	1.74	0.95 – 3.17	0.93	0.43 – 2.00
**Term**	Normal BW	1		1		1		1	
	LBW	2.95	2.75 – 3.16	3.04	2.81 – 3.29	3.72	3.36 – 4.13	2.62	2.09 – 3.27

## Discussion

One out of five newborns at MNH had LBW. There was also a high incidence of stillbirths and early neonatal deaths within 24 hours. LBW babies were more likely to die before birth, during labour and within 24 hours as compared to normal-weight babies. The LBW rate (21%) found in this study was higher than that reported in similar studies globally (which is 15% overall and 16.5% in developing countries) [2]. It was also higher than that reported in northern Tanzania [6] and other Eastern African countries [2]. The reasons for these differences might be due to MNH being the highest referral point in Tanzania, commonly caring for complicated pregnancies. Furthermore, mothers whose babies are born preterm, and those affected by PROM and hypertension, might be candidates for planned early deliveries due to the routine practice of conducting CS for the prevention of adverse newborn and maternal outcomes. In this study, LBW was more prevalent in those mothers who lived in semi-urban areas. The urban residence could be a reflection (proxy indicator) of good economic status of the mothers, which is associated with normal birth weight, reflecting good maternal nutrition and wellbeing, both of which have been reported to lead to good birth weights [13]. Also, those residing in urban settings are more likely to have good physical accessibility to the health facilities affecting ANC attendances [20,21]. As reported in other studies [6,13,14], LBW was associated with women who had a lower level of education. This may be explained by an increased awareness among educated women in relation to available health services and their increased likelihood to attend ANC earlier and more frequently [21]. Women who attended less than 4 ANC visits had increased odds of delivering LBW babies. Similar findings have been reported elsewhere in the literature [3,6,22,23]. The main documented reasons are that a lack of focused ANC visits is associated with inadequate/failure to receive proper nutritional education during pregnancy and failure to obtain vitamin and mineral supplementations, such as folic acid and iron tablets, as well as the identification and management of infections [24]. In this study, teenage pregnancy was associated with higher rates of LBW (27.4%). This finding is like those of other studies conducted elsewhere [2,3,6,14,25]. Some reasons for these results could be related to the fact that pregnant teenagers are more likely to originate from poor families and lack good education, which are strong risk factors for LBW [26].

Teenage pregnancies are also more likely to be primigravida, whereby the first pregnancy has higher odds of the mother delivering an LBW baby compared to subsequent pregnancies [21]. The findings from this study show that single mothers gave birth to more LBW babies than married mothers. Economic and psychosocial support could be a key factor contributing to good maternal health and birth outcomes [6]. In this study, mothers who had anaemia during pregnancy had nearly three times as high odds (OR: 2.9) of having LBW babies as those reported by Oladeinde et al in Nigeria [21]. As reported in other studies, anaemia is a proxy indicator of poor nutrition during pregnancy, which gives rise to IUGR [12,27]. In the regression model we found no effect of HIV infection on LBW, unlike other reports [22,28]. This could be due to a long-term result of effective and well-implemented PMTCT interventions comprising the provision of ARVs, frequent follow-up visits for care, and adequate drug refills, and vitamin and other nutritional supplements among HIV-infected pregnant women [29,30]. In this study we found a strong association between LBW and gestational age (GA), whereby low GA increased odds of LBW. The main reason that has previously been attributed to that association is the level of prematurity of the babies [4,6,21]. Only a third of the LBW babies in this study were premature, suggesting that the rest had IUGR. However, multivariate regression analysis showed that both prematurity and IUGR independently predicted LBW. Furthermore, separate regression analysis revealed that, in both premature and term deliveries; LBW predicted adverse perinatal outcomes. These findings suggest that interventions should target both prematurity and LBW. IUGR might have been caused by maternal illnesses such as anaemia and hypertensive disorders, and poor maternal nutrition [31] during pregnancy, hence restricting the growth of the fetus [32,33]. We found that LBW was strongly associated with poor immediate and 24-hour birth outcomes. Several reports have also associated LBW with poor perinatal outcomes, including stillbirth, low Apgar score, and death within 24 hours after birth [2,7,8]. One reason for these adverse fetal outcomes in a majority of mothers is complications related to prematurity, and hence their newborns are more likely to have Respiratory Distress Syndrome (RDS), are easily hypothermic due to immature thermoregulatory centers, tend to lose more energy due to their large surface area to volume ratio, have breathing difficulties due to immature lungs (inadequate surfactant) and are likely to develop infections. There is a need for concerted intervention (steroids, antibiotics and temperature management such as kangaroo mother care) against these identified factors [34-37].

**Strength of the study**: This study used a large database that has been designed and monitored continuously over a lengthy period by experienced researchers [17,18,28].

**Limitations of the study**: First, this is a hospital-based study; hence the prevalence of LBW is high, as MNH is a tertiary health facility that receives women affected by complicated pregnancies and deliveries, most of whom live in Dar es Salaam and its neighboring regions. Secondly, there was a significantly substantial number of clients with incomplete data (nearly 20%), but we believe that the sample size was still large enough to make the results internally valid.

## Conclusion

The prevalence of LBW was high and was independently associated with extreme maternal age groups, grand multiparity, low maternal education, low ANC visits, and obstetrics risks factors and complications. Both LBW and prematurity were independently associated with poor perinatal outcome. Future interventions should focus on improving quality of ANC and integrating peripartum emergency obstetric and neonatal care to prevent LBW and improve the survival rate.

### What is known about this topic

- There is an unmet demand of health resources for care of low birth weight in sub-Saharan Africa, including Tanzania, that cares for more than 90% of low birth weight newborn globally;
- Low birth weight is associated with poor perinatal outcomes and morbidity in childhood and adulthood, however, limited literature addresses risk factors, disease burden and complications of low birth weight;
- Antenatal risk factors such as hypertension and premature rupture of membranes, antenatal screening, are independently associated with both low birth weight and poor perinatal outcome.

### What this study adds

- The prevalence of low birth was found to be even higher at tertiary heath facility demand for prioritizing health resource for care of low birth weight and premature new-born at referrals hospitals.Low birth weight leads to poor perinatal outcomes demands for the quality of antenatal and peripartum care at tertiary health facilities;
- A feedback loop of motivators and challenges for meeting standards for antenatal screening and Emergency Obstetric and Neonatal Care intervention need to be integrated in the Reproductive Maternal Newborn and Child Health policy;
- The use of routine hospital statistics as a means of identifying target areas for quality improvement in maternal and newborn care within the referral chain.

## Competing interests

The authors declare no competig interests.
